# Antibacterial and anticancer potential of bioactive compounds and secondary metabolites of endophytic fungi isolated from *Anethum graveolens*

**DOI:** 10.3389/fmicb.2024.1448191

**Published:** 2024-10-07

**Authors:** Hoda R. A. El-Zehery, Noha Mohamed Ashry, Abeer A. Faiesal, Mohamed S. Attia, Mostafa A. Abdel-Maksoud, Mohamed A. El-Tayeb, Mohammed Aufy, Noha K. El-Dougdoug

**Affiliations:** ^1^Department of Agricultural Microbiology, Faculty of Agriculture, Benha University, Benha, Egypt; ^2^Department of Basic and Applied Agricultural Sciences, Higher Institute for Agriculture Cooperation, Cairo, Egypt; ^3^Botany and Microbiology Department, Faculty of Science, Al-Azhar University, Cairo, Egypt; ^4^Department of Botany and Microbiology, College of Science, King Saud University, Riyadh, Saudi Arabia; ^5^Department of Pharmaceutical Sciences, Division of Pharmacology and Toxicology, University of Vienna, Vienna, Austria; ^6^Department of Botany and Microbiology, Faculty of Science, Benha University, Benha, Egypt

**Keywords:** endophytic fungi, medicinal plants, *Anethum graveolens*, antitumor, secondary metaabolites

## Abstract

Fungal endophytes are known to produce bioactive chemicals and secondary metabolites that are often identical to those produced by their host plants. The main objective of the current study was to isolate and identify endophytic fungi associated with the medicinal plant *Anethum graveolens*, and to investigate their potential antibacterial and anticancer properties. The ethyl acetate extracts from the isolated endophytic fungi, as well as the host plant *A. graveolens*, were subjected to bioactivity assays to evaluate their antibacterial and anticancer potential against multi-drug resistant bacterial strains and the human hepatocellular carcinoma cell line HepG2. The endophytic fungi isolated and identified from the *A. graveolens* samples included *Diaporthe, Auxarthron, Arthrinium, Aspergillus, Microsporum, Dothiorella, Trichophyton, Lophiostoma, Penicillium,* and *Trichoderma* species. The minimum inhibitory concentration (MIC) assay revealed that the *A. graveolens* extract exhibited the strongest antibacterial activity, with an MIC value of 4 μg/ml, followed by the *Trichoderma* sp. (5 μg/ml) and *Penicillium* sp. (6 μg/ml) extracts. Additionally, the crude extracts of *Trichoderma* sp., *Penicillium* sp., and *Fusarium* sp. demonstrated high anticancer activity against HepG2 cells, with inhibition rates ranging from 89 to 92% at a concentration of 50 μg/ml. Interestingly, the *A. graveolens* extract showed the most potent anticancer activity, with a 95% inhibition rate against HepG2 cells at the same concentration. These findings highlight the significant potential of endophytic fungi associated with *A. graveolens*, as a source of bioactive compounds with promising antibacterial and anticancer properties. The results reinforce the hypothesis that medicinal plants and their endophytic fungi can serve as an attractive alternative for the development of novel therapeutic agents, potentially offering a more sustainable and less harmful approach to disease management compared to traditional chemical-based methods.

## Introduction

Each of the nearly 300,000 distinctive terrestrial plant species has the potential to harbor one or more of the millions of fungi that can serve as hosts for these plants (Rashmi et al., 2019). Endophytes are microorganisms that can exist dormant within the host’s tissues for an extended period without manifesting any outward symptoms of sickness (Mishra et al., 2021). They have the potential to be used as a biocontrol agent, which is becoming the preferred approach to disease management because of the various positive effects that it has on both human health and the preservation of the natural environment (Lahlali et al., 2022). They have the potential to be used as a biocontrol agent (Javaid et al., 2021; Ons et al., 2020). Endophytes have a variety of mechanisms, such as the capability to activate specific genes that are involved in induced systemic resistance (ISR), which can initiate a defense mechanism against an attack by pathogens, or the capability to formulate secondary metabolites and other chemical compounds that are directly toxic to the pathogens (Anjum et al., 2019; Khan et al., 2021). Endophytes are found in all plant species regardless of their place of origin. The ability to enter and thrive in the host tissues makes them unique, showing multidimensional interactions within the host plant. Several host activities are known to be influenced by the presence of endophytes. They can promote plant growth, elicit defense response against pathogen attack, and can act as remediators of abiotic stresses (Khare et al., 2018). The variety of fungi that can be found on or within the host plant is significantly influenced not only by the type of plant that acts as the host but also by the environmental conditions under which the plant grows or the geographical elements that are present (Dastogeer et al., 2020). According to the findings of the Sofy et al. (2020) study, endophytic fungal strains were collected from a wide range of plants. These plants included vegetables, fruits, fodder, cereal grains, trees, and other types of crops. In addition, endophytes are a rich and dependable source of genetic variation as well as biological innovation, and they have been employed in agriculture as well as pharmacology (for example, in medicines that combat cancer, fungi, viruses, and bacteria; Fontana et al., 2021).

Medicinal plant extracts may contain hundreds or even thousands of individual biologically active compounds in varying amounts that enable them to treat a wide range of conditions, the overall activity of medicinal plant extracts is the result of the combined action of multiple compounds with synergistic, additive or antagonistic activity (Vaou et al., 2022).

The plant species *Anethum graveolens*, more often referred to as dill, was chosen for the purpose of this inquiry (Sulieman et al., 2023). This plant belongs to the family *Apiaceae*, which also includes celery and parsley (Wang et al., 2022). The *A. graveolens* plant has been linked to having actions that include antioxidant, anti-inflammatory, anti-diabetic, antibacterial, and anticancer (Ahmed et al., 2021). It has been established that extracts of *A. graveolens* possess significant antioxidant activity against fungi that are toxic to both plants and people (Al-Oqail and Farshori, 2021). The unchecked multiplication of cells, which ultimately leads to the creation of a tumor, is one of the defining characteristics of cancer (Compton and Compton, 2020). These cells are not the same as regular cells and do not participate in the standard mechanisms that regulate growth (Meng et al., 2006). According to Chen et al. (2015), the cytotoxic and anti-proliferative capabilities of plants and plant-derived components can be examined using a range of cancer cell lines by employing a number of different cytotoxic endpoints. These endpoints include MTT, neutral red uptake, and cellular morphology investigations (Cudazzo et al., 2019). Plants produce a substantial quantity of active components, and in comparison to other sources, these components from plants are less harmful (Alamgir, 2017). Buranrat et al. (2020) stated that one of the current trends in the fight against cancer is the quest for an anticancer agent derived from plants that have the power to both halt and reverse the progression of the disease.

Consequently, the objective of this research was to explore the biological activity (antibacterial and anticancer) of *A. graveolens* and endophyte fungi extracts against human cancer cells and multi-drug-resistant pathogenic bacteria, as well as to isolate and identify colonized endophytic fungi. Furthermore, the investigation endeavored to isolate and identify colonized endophytic fungi.

## Materials and methods

### Collection of plant

Matured healthy *Anethum graveolens* plant was collected from the farm of the Horticulture Department, Faculty of Agriculture, Ain Shams University. The plant’s stem, root, and leaves were cut off using a sterile knife, and they were then preserved in sterile plastic bags at 4°C until usage.

### Endophytic fungi isolation

According to Lu et al. (2012) each sterile part was placed on potato dextrose agar (PDA) supplemented with penicillin and / or streptomycin (3 mg/100 ml) and incubated at 28°C. On Sabouraud agar, the fungal hyphae tips were inoculated and incubated for 7 days at 24 until the selected single distinct colony morphotype (Zhang et al., 2019). The pure cultures isolates were maintained at 4°C on Sabouraud agar slant tubes.

### Identification of endophytic fungi isolates

All isolates were described depending on their morphological characteristics (Barnett and Hunter, 1998; Hashem et al., 2019; Khalil et al., 2013; Khalil et al., 2019; Watanabe, 2010). Macroscopic morphological features including color, texture and diameter of colonies and microscopic characteristics including vegetative and reproductive structures of the fungi were noted.

### Multi-drug pathogenic bacterial strains and culture conditions

*Staphylococcus aureus* (ATCC 6538) *Bacillus cereus* (ATCC9634), *Escherichia coli* (ATCC10536)*, Pseudomonas aeruginosa* (ATCC10145)*, Salmonella typhi* (ATCC 8435) were obtained from the Cairo MARCN Fac. of Agric. Ain Shams Univ. Egypt. Twenty-four hours before each experiment, bacteria were sub cultured on nutrient agar tubes at 37°C. These strains were used for the bioactive study of mycelia and culture broth extracts of isolated endophytic fungi.

### Bioactivity assay

#### Extraction of endophytic fungi isolates

Each isolate was cultivated in a 500 ml Erlenmeyer’s flask containing 100 ml of Sabouraud broth at a pH 5.6, inoculated by fungal mycelium, and cultivated for 7 days at 220 rpm in an incubator shaker (Chathurdevi and Gowrie, 2016). From fermentation, fungal mycelia and broth medium were obtained. At 35°C, the solvent was combined and concentrated in a vacuum. The crude extracts were kept at −20°C until analysis.

#### Extraction maceration of *A. graveolens* plants

The 10 g of extracted plant material were shaken and stirred irregularly for 3 days while submerged in ethyl acetate (1:2 W/V) in an airtight, flat-bottomed jar. In a cell disintegrator, the sterile plant material was air-dried and homogenized, then extracted with ethyl acetate (1:2 W/V). Using 2 thicknesses of cheesecloth, the extraction was filtered. At 35°C, in a vacuum, the solvent was mixed and concentrated (Ibrahim et al., 2021).

#### Minimum inhibitory concentration determination

The inhibitory effects of 11 endophyte fungus extracts against five pathogenic bacterial strains were assessed by the microdilution method (Ibrahim et al., 2021). First, each well received 50 μl of the crude extract of each isolated fungus and 100 μl of the pathogenic bacteria culture (1*10^8^ CFU/ml). The harmful bacterial strains were cultivated for 48 h at 37°C. Then, using an absorbance value near to the blank value and no discernible turbidity in the pores as the standard, the minimum inhibitory concentration (MIC) of the chemical was calculated at 600 nm. The positive control for antibacterial was an antibiotic (ampicillin). Three repetitions were planned for each test. The microbial growth reduction percentage (GR %) was calculated using the broth microdilution method with the control treatment as the guideline as $GR\%=\frac{C-T}{Cx100}$ .

Where, T = replicates, C = cell concentrations under the control treatment. The outcomes of the experiment were recorded as mean ± SE of the triplicate samples (NCCLS/CLSI, 2015).

### Biofilm-disruption assay

The tested multi-drug pathogenic bacterial strains were inoculated with 50 μl in nutrient broth media in 96-well microtiter and incubated for 72 h at 30°C to allow biofilm formation on the well (Dusane et al., 2010). Each endophytic fungal isolate extract was poured into microtiter wells to determine anti-disruptive activity. Planktonic cells were removed after 24 h. of incubation and washed twice with PBS. To evaluate absorbance at 595 nm, adherent bacteria were fixed in methanol (99%) and stained with crystal violet. Pathogen biofilms in wells not treated with extracts were used as controls. Biofilm disruption activity was measured as the percentage of biofilm formed in extracts-treated wells compared to untreated biofilms (control negative) and treated with (ampicillin) control positive.

### Cytotoxicity activity (MTT assay)

The company for Biological Products and Vaccines (VACSERA) supplied the monolayer microtiter plate with HepG2 cell monolayers. MTT assay is a colorimetric assay that relies on intracellular mitochondrial succinate dehydrogenase enzymes in metabolically active cells to convert the yellow, With certain adjustments, according to (Mosmann, 1983). For a 48-h exposure period, cell monolayers were treated in quadrature with test IC50 for each extract. On a Tristar LB2 microplate reader (Berthold, Germany), the absorbance was measured at 492 nm against a blank (no cells) after adding DMSO (100 μl/well) to dissolve the formazan crystals for 10 min of shaking. The following formula was used to determine the viability percentage: Inhibiting cells (%) = 100 − Surviving cells; % viability = (At − Ab)/(Ac − Ab)100.

## Results

### Eleven fungal endophytes isolation and identification

**Figure 1:**
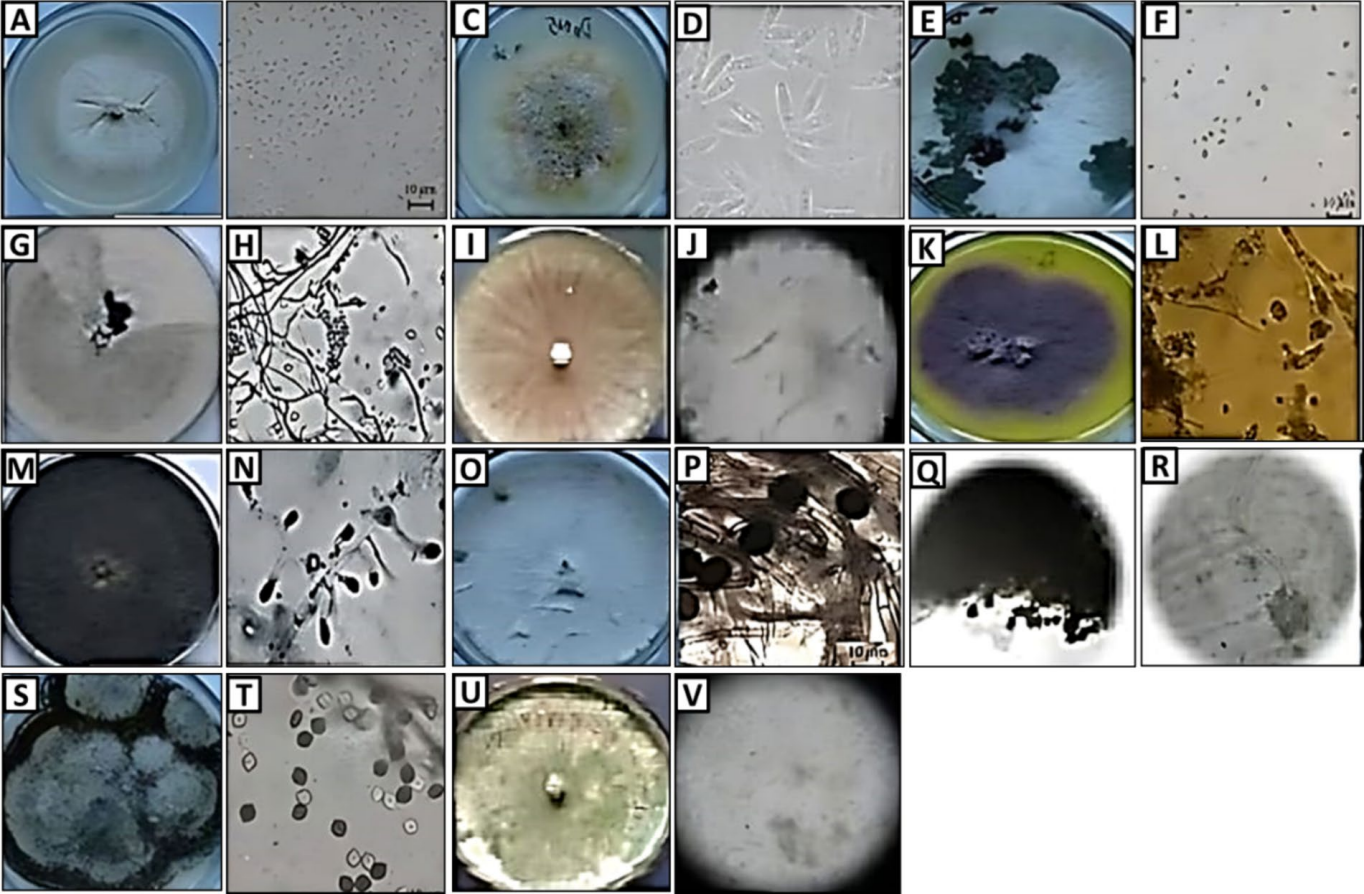
Morphology (colony appearance, conidia, and hypha) of endophytic fungi: *Arthrinium* sp. **(A,B)**, *Auxarthron* sp. **(C,D)**, *Dothiorella* sp. **(E,F)**, *Trichoderma* sp. **(G,H)**, *Fusarium* sp. **(I,J)**, *Penicillium* sp. **(K,L)**, *Trichophyton* sp. **(M,N)**, *Microsporum* sp. **(O,P)**, *Aspergillus* sp. **(Q,R)**, *Lophiostoma* sp. **(S,T)**, and *Diaporthe* sp. **(U,V)**.

Eleven fungal endophytes were isolated from *A. graveolens* and identified according to morph type characteristics, colony appearance, reverse colony, and microscopic conidia. The isolated endophytes fungi belonged to nine different families, *Trichocomaceae*; *Onygenaceae; Apiosporaceae; Diaporthaceae; Hypocreaceae; Nectriaceae; Lophiostomataceae; Botryosphaeriaceae* and *Trichocomacea*. The 11 fungal endophytic were *Aspergillus* sp. *Trichoderma* sp. *Penicillium* sp. *Dothiorella* sp. *Arthrinium* sp. *Fusarium* sp. *Diaporthe* sp. *Microsporum* sp. *Auxarthron* sp. *Trichophyton* sp. and *Lophiostoma* sp. The most abundant fungi were *Trichoderma* sp. (16.3%)*, Penicillium* sp. with (14.6%)*, Aspergillus* sp. (11.6%)*, Dothiorella* sp. (10.5%), *Arthrinium* sp. (8.3%), *Diaporthe* sp. with (8.0%)*, Auxarthron* sp. (7.6%), *Fusarium* sp. (7.3%), *Trichophyton* sp. (6.4%), *Microsporum* sp. (4.9%) and *Lophiostoma* sp. with (4.3%; Figure 1).

### Antibacterial activity

Regarding the antibacterial activities, percentage of microbial growth inhibition (GR) ranged from 10.2 to 18.8%, mean growth inhibition (MGI) ranged from 0.24 to 0.87 OD, and minimum inhibitory concentrations (MIC) ranged from 10 to 42.5 (μg/ml) against all tested multi-drug pathogenic bacterial strains under research. The strongest antibacterial activity was demonstrated by *A. graveolens, Trichoderma* sp. and *Penicillium* sp. extracts. Additionally, data showed that *Microsporum* sp. exhibited the lowest antibacterial activity against Gram-positive *B. cereus* and *S. aureus*. While, *Aspergillus* sp. against Gram-negative *E. coli, P. aeruginosa*, and *Salmonella typhi* (Table 1).

**Table 1:** *A. graveolens* and fungal endophytes extract against selected multi-drug pathogenic bacterial strains.

Extracts	*B. cereus* (ATCC9634)			*E. coli* (ATCC10536)			*P. aeruginosa* (ATCC10145)			*Salmonella typhi* (ATCC 8435)			*S. aureus* (ATCC 6538)		
	GR%	MGI	MIC	GR%	MGI	MIC	GR%	MGI	MIC	GR%	MGI	MIC	GR%	MGI	MIC
*Arthrinium* sp.	12.6^g^	0.62^b^	25^b^	13.4^h^	0.58b	20e	14.7d	0.64c	15d	13.3d	0.50c	20e	14.5d	0.51e	25c
*Aspergillus* sp.	13.4^f^	0.69^ab^	15^d^	13.4^h^	0.54^b^c	25d	12.1i	0.55d	15d	10.2h	0.25d	26.9b	13.5f	0.45g	25c
*Auxarthron* sp.	14.4^d^	0.48^c^	20^c^	13.5^h^	0.50c	20e	14.3e	0.42e	15d	13.2d	0.57c	18.4f	13.7e	0.49f	24c
*Diaporthe* sp.	13.4^f^	0.69^ab^	25^b^	14.3^f^	0.75a	40a	13.6g	0.64c	25b	12.4f	0.55c	25.8c	14.6d	0.62c	25c
*Dothiorella* sp.	14.5^cd^	0.60^b^	25^b^	12.6^i^	0.25d	25d	11.5j	0.75b	25b	11.6g	0.75b	20.0e	13.5f	0.55d	25c
*Microsporum* sp.	11.3^h^	0.32^d^	20^c^	14.7^e^	0.24d	30c	14.6d	0.42e	35a	12.3f	0.50c	18.2f	12.5h	0.50^ef^	15e
*Fusarium* sp.	12.5^g^	0.69^ab^	15^d^	12.5^i^	0.55^b^c	35b	12.4h	0.55d	25b	13.2d	0.72b	16.9g	13.7e	0.51e	30b
*Trichophyton* sp.	14.1^e^	0.45^c^	20^c^	13.8^g^	0.57^bc^	20e	13.8f	0.64c	20c	12.6e	0.56c	42.5a	12.7g	0.49f	30b
*Lophiostoma* sp.	12.5^g^	0.62^b^	25^b^	14.9^d^	0.55^bc^	25d	14.6d	0.85a	15d	11.5g	0.50c	22d	13.6ef	0.46g	35a
*Penicillium* sp.	14.6^c^	0.70^ab^	30^a^	15.6^c^	0.58b	20e	15.2c	0.86a	25b	15.5c	0.73b	15h	15.2c	0.65b	20d
*Trichoderma* sp.	16.8^b^	0.80^a^	15^d^	16.5b	0.59b	15f	15.6b	0.85a	15d	15.8b	0.77b	12.2i	16.2b	0.63c	15e
*A. graveolens extract*	18.7^a^	0.81^a^	10^e^	17.8a	0.80a	10 g	16.9a	0.87a	10e	18.8a	0.85a	10j	17.4a	0.71a	10f
Ampicillin (50 mg)	0.0^i^	0.4^cd^	0.0^f^	0.0j	0.5c	0.0h	0.0 k	0.2f	0.0f	0.0i	0.20d	0.0l	0.0i	0.39h	0.0g

Each value is the mean of 3 replicates.GR % = the percentage of microbial growth reduction; MICs (μg/ml) = Minimum Inhibitory concentrations; MGI (OD) = Mean growth inhibition. At *p* 0.05, the same column’s distinct letter combinations in letters a, b, c, d, e, f, g, h, and i are significantly different.

Metabolite broths of all 11 endophyte fungi isolate extract were growth reduction of tested pathogenic bacteria with ranged from 11.3 to 16.8 (*B. cereus*), 12.5–16.5 (*E.coli*), 11.5–15.6 (*P. aeruginosa*), 10.2–15.8 (*Salmonella typhi*) 12.5–16.2 (*S. aureus*) as well as *A.graveolens* extracts, 18.7, 17.8, 16.9, 18.8, and 17.4, respectively compared with ampicillin (50 mg). The mean growth inhibition was recorded in all tested multi-drug pathogenic bacterial strains ranging from 0.32 to 0.81, 0.24 to 0.80, 0.42 to 0.87, 0.25 to 0.85, and 0.45 to 0.71 OD for *B. cereus*, *E. coli*, *P. aeruginosa*, *Salmonella,* and *S. aureus,* respectively by broth micro-dilution method compared to ampicillin (50 mg) was 0.4, 0.5, 0.2, 0.2, 0.39 OD. The MIC values were recorded against tested pathogenic bacterial strains ranging from 10 to 42.5 μg/ml. Generally, results showed that the MIC values of *A. graveolens* extract were the lowest followed by *Trichoderma* sp. then other endophyte fungi isolates.

### Biofilm disruption

The percentage of biofilm reduction of ethyl acetate extracts from plant tissues and isolated fungal endophytes compared to the antibacterial drug ampicillin All tested pathogenic bacteria have biofilm formation with different values of 0.80, 0.75, 0.95, 0.82, and 0.85 with optical density (OD) for *B. cereus, E.coli, P. aeruginosa, S. typhi*, and *S. aureus,* respectively (Table 2).

**Table 2:** Biofilm disruption assay of ethyl acetate extract for fungal endophytes and *A. graveolens* compared to ampicillin.

Organisms extracts	Biofilm formation (OD)				
	*B. cereus* (ATCC9634)	*E. coli* (ATCC10536)	*P. aeruginosa* (ATCC10145)	*Salmonella typhi* (ATCC 8435)	*S. aureus* (ATCC 6538)
Biofilm formation (OD)	0.80	0.75	0. 95	0. 82	0.85
Biofilm reduction (%)					
Arthrinium sp.	75c	47f	55e	32g	75c
*Aspergillus* sp.	60g	65c	75b	52d	45g
*Diaporthe* sp.	65f	75b	65c	81.8a	65d
*Auxarthron* sp.	73d	45g	85a	72b	81b
*Dothiorella* sp.	72d	65c	75b	62c	45g
*Microsporum* sp.	60g	55e	59d	48e	58e
*Fusarium* sp.	57h	65c	45f	52d	45g
*Trichophyton* sp.	57h	57d	59d	51d	54f
*Lophiostoma* sp.	59g	55e	45f	42f	65d
*Penicillium* sp.	68e	65c	65c	72b	80b
*Trichoderma* sp.	77b	65c	75b	72b	65d
*A. graveolens*	80a	85a	85a	82a	85a
Ampicillin (50 mg)	25i	15h	24g	15h	25h
MSE	4.25	5.1	3.65	4.53	4.63
*p*-value	<0.0001	<0.0001	<0.0001	<0.0001	<0.0001

At *p* 0.05, the same column’s distinct letter combinations in letters a, b, c, d, e, f, g, h, and i are significantly different.

Regarding extract of fungal endophytes, showed significant anti-biofilm activity against *B. cereus*, *E.coli*, *P. aeruginosa*, *S. typhi*, and *S. aureus* with an inhibition rate ranging between 57 to 77%, 45 to 75%, 45 to 85%, 42 to 82%, and 45 to 81%, respectively. Also, results indicated that the highest anti-biofilm activity was achieved by *A. graveolens* extract significantly inhibited the biofilm formation of *B. cereus, E.coli, P. aeruginosa*, *S. typhi*, and *S. aureus* with 80, 85, 85, 82, 85%, respectively. However, ampicillin (50 mg) showed the lowest anti-biofilm activity with a value of 25, 15, 24, 15, and 25% for *B. cereus, E.coli, P. aeruginosa*, *S. typhi*, and *S. aureus*, respectively.

### Anti-tumor activity

**Figure 2:**
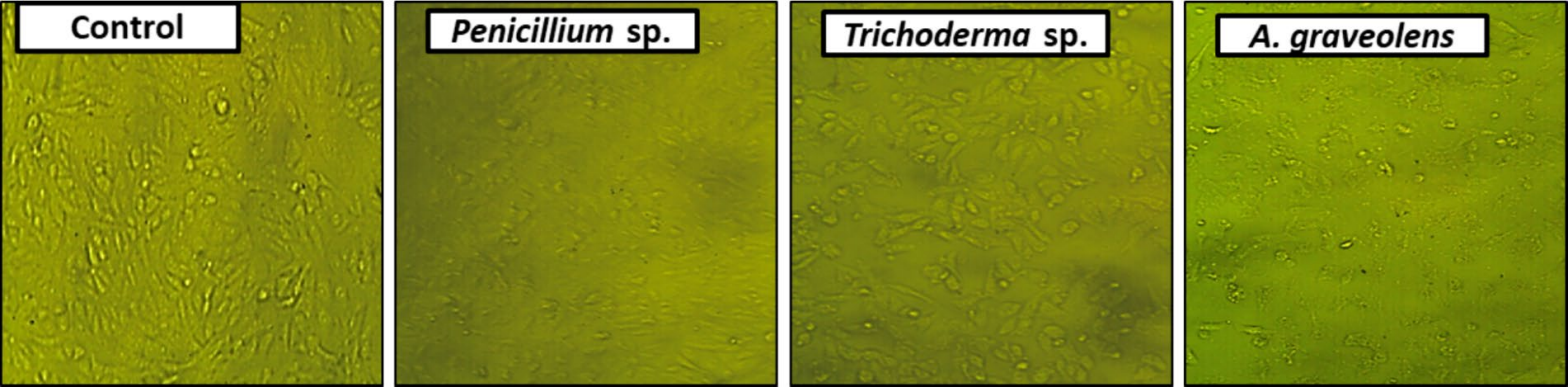
Photomicrographs displaying alterations in HepG2 monolayer morphology following treatment with a specific concentration of *Penicillium* sp. *Trichoderma* sp. or Plant (*A. graveolens*) extracts, as well as a control (0.5% DMSO). Monolayer rupture, cell rounding, cell contraction, and/or cell lysis are symptoms of cytotoxicity.

In the initial screening of 11 endophytic fungi and plant extracts for antitumor activities, the extracts of *A. graveolens*, *Trichoderma* sp. and *Penicillium* sp. displayed the greatest anticancer activity against human HCC (HepG2) compared to the control (0.5% DMSO). The morphology of treated cells exhibited symptoms of toxicity, involving shrinkage, cell rounding, and/or monolayer rupture representing effects on HepG2 cells. At (50 μg/ml) the crude extract of *Trichoderma* sp. was the best anticancer activity against HepG2 cells among the studied extracts. Plant extract > *Penicillium* sp. > *Trichoderma* sp. are the most active fungal endophytic extracts against the HepG2 tumor (Tables 3, 4; Figure 2).

**Table 3:** Endophytic fungi ethyl acetate and *A. graveolens* extract and their anticancer activity against (HepG2) cells.

Extracts μg/ml	Concentration μg/ml					
	IC_50_	50	40	30	20	10
	Cell inhibition %					
*Arthrinium* sp.	4	80.75d	70.75g	65.85d	45.75g	30.75d
*Aspergillus* sp.	5	85.25c	70.25h	65.45d	45.25g	30.25d
*Diaporthe* sp.	5	82.00d	75.65e	70.12c	52.00e	25.65e
*Auxarthron* sp.	5	81.00d	73.36f	65.56d	50.00f	23.36f
*Dothiorella* sp.	5	90.00b	75.60d	63.00e	50.25f	35.00c
*Microsporum* sp.	5	90.75b	80.75c	65.85d	52.75e	40.75b
*Fusarium* sp.	3	89.25b	76.25d	65.45d	55.25 d	42.25g
*Trichophyton* sp.	5	85.00c	75.65e	65.12d	54.10d	35.65c
*Lophiostoma* sp.	5	90.00b	75.75d	75.56b	62.30b	23.36f
*Penicillium* sp.	6	92.75b	83.36b	68.45d	68.00a	40.75b
*Trichoderma* sp.	5	90.00b	76.25d	65.85d	60.75c	40.00b
*A. graveolens*	4	95.25a	86.25a	80.00a	75.25a	50.25a

IC_50_ = the half-maximal inhibitory concentration. At *p* 0.05, the same column’s distinct letter combinations in letters a, b, c, d, e, f, g, and h are significantly different.

**Table 4:** Morphological and cultural characteristics of selected *A. graveolens* endophytic fungi.

**Fungal isolate**	**Colony appearance**	**Reverse colony color**	**Microscopic conidospores**	**Frequency**	
				**Sponge (CFU/g)**	**%**
Family: Apiosporaceae *Arthrinium* sp.	Smooth, hyaline, branching, septate. Reduced, pale brown, smooth, ampulliform Conidiophores; brown, smooth, globose Conidia.	Brownish	Conidiogenous cells form clusters on hyphae and are pale brown and ampulliform.	125	8.3
Family: Trichocomaceae *Aspergillus* sp.	Blackish-brown, globose conidial heads on long, erect, hyaline conidiophores were a distinguishing feature of this fungus. Conidia were roughly round, dark brown, rough, or echinulate.	Blackish	conidiophores had typical conidial heads on long,	175	11.6
Family: Diaporthaceae *Diaporthe* sp.	The white mycelium became a greyish-white hue. Individual, semi-submerged pycnidia.	White	Conidiomorphs Alpha conidia were hyaline, smooth, ellipsoidal, and had a base that was subtruncate. and beta conidia were hyaline,	120	8.0
Family: Onygenaceae *Auxarthron* sp.	Make globose gymnothecia with branched hyphae, spiny apices, and extended appendages.	Brown	Ascospores areglobose and punctate to punctatereticulate. Anamorphs are distinct	115	7.6
Family: Botryosphaeriaceae *Dothiorella* sp.	Conidiomata with thick walls, pycnidial, globose, superficial or semi-immersed, hyphal hair-covered surfaces, and unilocular structures. Hyaline subcylindrical to ellipsoid spores brown.	Bluishviole	No conidiophores are seen.	158	10.5
Family: Hypocreaceae *Microsporum* sp.	Buff with irregular branching hyphae with conspicuous cross walls (bamboo hyphae) (bamboo hyphae)	Red	the greater size, multiseptate, oval shape, and extremely thick wall (5 cells) Clavate	75	4.9
Family: Nectriaceae *Fusarium* sp.	Pale to bluish violet / orange, incredibly slimy pin notes, full of macroconidia produced borne on monophialide in False heads oblong, straight, septate, and when old pion notes turned brown.	Bluishviole	Abundantly produced microconidia vary greatly in size and shape (Kidney-shaped). Chlamydospores are solitary and terminal on short lateral branches.	110	7.3
Family: Hypocreaceae *Trichophyton* sp.	White with a downy surface and growing suede-like to downy Pleomorphic projections.	Brown	Thin-walled, cigar-shaped, and multi-celled. Numerous pyriform smooth-walled single cells with spiral hyphae	96	6.4
Family: Lophiostomataceae *Lophiostoma* sp.	A body shape with a small to big flat, crest-like tip that can range from globose to subglobose. Single-layered, composed of tiny, weakly pigmented, thin-walled cells, with pseudo parenchymal cells constituting the apex.	Black	The hamathecium is lengthy and dense, septate, covered in mucilage, anastomosing, branching, bitunicate, fissitunicate, clavate, pedicellate, and it has a little ocular chamber. Hyaline ascospores	65	4.3
Family: Trichocomaceae *Penicillium* sp.	Colonies color daylight-visible mycelium bluish green to dark green and conidia greenish. Colonies bluish green or a mixture of green and grey, turning brownish with age, cottony to sub-floccose, broadly spreading, and with a substantial sterile margin when young. The majority of conidiophores grow autonomously. Conidia elliptical, turning globose, pale green.	Yellow	Conidiophores arising from the mycelium singly or less often in synnemata, branched near the apex stipe long or short, penicillate, from monoverticillate to teliverticillate, terminating in a group of phialides; conidia (phialo spores) hyaline or brightly colored in mass, celled, predominantly globose or avoid predominantly spherical or subs	220	14.6
Family: Hypocreaceae *Trichoderma* sp.	The growth of one was yellowish-green in color. The heads are radiated and yellowish-green. The stip was long. The vesicle was globose and absent of meulae. The conidia were absent and globose very rough, and greenish. Colonies at first white later becoming greenish. Pyramidal.	Greenish	Conidiophores are extensively branched, with branches originating from the hyphae of origin. Conidia were pale green, smooth, globose or oval, unicellular, and with thin walls. Phialides were flask-shaped and grouped in 2-4 distinct groupings. The conidiophore system is observed	245	16.3

## Discussion

Fungal endophytes are fungi that live within plant tissues without harming the host. They can enhance plant defense, stress tolerance, and nutrient acquisition (Badawy et al., 2021; Sharaf et al., 2022). Endophytes form symbiotic relationships, benefiting both the host plant and the fungus. Understanding fungal endophytes has applications in agriculture, forestry, and natural product development (Abdelaziz et al., 2022a; Abdelaziz et al., 2022b; Attia et al., 2022). In this study, 11 endophyte fungi were isolated from multipurpose plant *A. graveolens*. The isolated fungal endophytes belonged to 9 different families: *Apiosporaceae, Trichocomaceae, Diaporthaceae, Onygenaceae, Botryosphaeriaceae, Hypocreaceae, Nectriaceae, Lophiostomataceae* and *Trichocomaceae*. The more frequent genus are *Aspergillus* sp. *Trichoderma* sp. *Penicillium* sp. and *Dothiorella* sp. Where the number and type of isolated fungi differ according to the plant variety and plant tissues. *Daldinia* sp. and *Lentinus* sp. were isolated from plant leaf tissue while *Rigidoporus* sp. and *Polyporales* sp. were from root tissue (Maadon et al., 2018). *Penicillium* sp. two of *Aspergillus* spp. and *Trametes hirsuta* were identified in coconut (Salo and Novero, 2020). *Fusarium chlamydosporum*, *Phoms* sp. *Fusarium oxysporum*, *Alternaria solani*, *Fusarium equiseti*, *Stemphylium* sp., 8 from leaf segments of *Avicennia marina* (Khalil et al., 2021). We observed that Dothiorella sp. which is isolated from salt-tolerant plant like Mangrove (Cadamuro et al., 2021) is among the more frequent endophytes in our target plant, this indicates to may be *A. graveolen* has microbe-plant interaction mechanisms for salt tolerance but this needs more investigation.

For the development of prospective cytotoxic and antibacterial medications, endophytic fungi of medicinal plants are useful sources of active natural compounds (Mohamed et al., 2022), these compounds may be secreted or not (Charria-Girón et al., 2021; Gu et al., 2022). Fungal endophytes are known to produce a variety of antimicrobial compounds that can inhibit the growth of pathogenic bacteria, fungi, and viruses (Elghaffar et al., 2022; Hashem et al., 2022). These antimicrobial metabolites help protect the host plant from infectious diseases, making endophytes a promising source for discovering new antimicrobial agents with potential applications in medicine and agriculture (Hashem et al., 2023).

In the current study, the 11 endophyte fungi secreted compounds that inhibited the tested pathogenic bacteria, *B. cereus*; *E.coli*; *S. aureus*, *S. typhi*; *P. aeruginosa*, with mean growth inhibition (MGI) ranging from 0.24 to 0.87 OD. In other studies, the inhibitory activity reached 96% of the total fungi isolated from *Artemisia argyi* namely, *Alternaria, Colletotrichum, Phoma, Diaporthe, Gibberella, Trichoderma, Chaetomium* and *Fusarium* against pathogenic microorganisms (Gu et al., 2022). Additionally, MIC values against the pathogenic bacterial strains that were examined ranged from 10 to 42.5 g/ml. *Fusarium oxysporum* extract, on the other hand, has demonstrated broad antagonistic activity against a wide spectrum of bacteria, with MIC values ranging from 0.156 to 5.0 mg/ml and MBC values ranging from 0.625 to 10.0 mg/ml. Additionally, it demonstrated suppression of *Saccharomyces cerevisiae* but not of *Candida albicans* and *Trichophyton interdigitale* (Chutulo and Chalannavar, 2020; Metwaly, 2019; Raina et al., 2018). It is important to note that we were successful in this work in inhibiting the pathogenic bacterial strains with low MIC values, demonstrating the potency of the compounds produced by the endophytic fungi isolates. Regarding biofilm disruption, the extract of fungal endophytes showed significant antibiofilm activity against five bacteria *B. cereus*, *P. aeruginosa*, *S. aureus*, *E. coli* and *S. typhi*. Whereas, many extracts of fungal endophytes showed antibiofilm activity against *Pseudomonas aeruginosa* only. This is evident in the following studies endophytic fungi, such as *Aspergillus nidulans* and *Alternaria alternata,* have excellent antibiofilm activity against pathogenic bacteria (Meenambiga and Rajagopal, 2018), *Alternaria alternate* showed anti-quorum sensing activity against *Pseudomonas aeruginosa* (Rashmi et al., 2018) and *Fusarium* sp. *Pestalotiopsis* sp. *Phoma* sp. *Aspergillus* sp. *Trichoderma* sp. *Penicillium* sp. *Phomopsis* sp. and *Colletotrichum* sp. *Chlamydomonas* sp. extracts showed significant inhibition in biofilm formation against *Pseudomonas aeruginosa* (Dawande et al., 2019; Nithya et al., 2014). Furthermore, we observed high levels of anticancer activity of crude extracts of *A. graveolens*; *Trichoderma* sp. and *Penicillium* sp. against (HepG2) cells with IC50 4, 5, and 6 μg/ml, respectively at concentrations of 50 μg/ml. According to studies, *Penicillium rubens, Alternaria alternata*, and *Aspergillus niger* extracts showed negligible cytotoxicity on normal human lung at concentrations up to 400 g/ml (Al-Rajhi et al., 2022). *Penicillium rubens* and *Aspergillus niger* extract exhibited inhibitory activity against prostate cancer proliferation with concentrations of 48 and 4.4 μg/ml, respectively, with mortality levels of 75.91 and 76.2% (Heydari et al., 2019). However, the crude extract of Aspergillus tubingensis up to 19 μg/ml showed strong antiproliferative activity against colon, hepatocellular, and breast carcinoma cell lines (Elkhouly et al., 2021). This study reinforced the hypothesis that medicinal plants and fungi isolated from them play an important role as anti-tumor compounds and offer an attractive alternative for disease management without the negative impact of chemicals.

## Conclusion

The present study demonstrates the successful isolation and identification of various endophytic fungi from the medicinal plant *Anethum graveolens*. These endophytic fungi, including *Diaporthe, Auxarthron, Arthrinium, Aspergillus, Microsporum, Dothiorella, Trichophyton, Lophiostoma, Penicillium, and Trichoderma*, were found to possess potent antibacterial and anticancer properties. The findings highlight the significant potential of these endophytic fungi as a source of bioactive compounds. The extracts from *Trichoderma* sp.*, Penicillium* sp.*, and Fusarium* sp. exhibited high anticancer activity against the human hepatocellular cancer cell line HepG2, with inhibition rates ranging from 89 to 92% at a concentration of 50 μg/ml. Interestingly, the extract from the host plant *A. graveolens* showed the most potent anticancer activity, with a 95% inhibition rate against HepG2 cells at the same concentration. These results reinforce the hypothesis that medicinal plants and their associated endophytic fungi can serve as a valuable resource for the discovery of novel therapeutic compounds, offering an attractive alternative to traditional chemical-based approaches for disease management.

## Data Availability

The original contributions presented in the study are included in the article/supplementary material, further inquiries can be directed to the corresponding author.
